# Stressors, Appraisal of Stressors, Experienced Stress and Cardiac Response: A Real-Time, Real-Life Investigation of Work Stress in Nurses

**DOI:** 10.1007/s12160-015-9746-8

**Published:** 2015-11-25

**Authors:** Derek Johnston, Cheryl Bell, Martyn Jones, Barbara Farquharson, Julia Allan, Patricia Schofield, Ian Ricketts, Marie Johnston

**Affiliations:** School of Psychology, College of Life Sciences and Medicine, University of Aberdeen, Kings College, Old Aberdeen, Aberdeen, AB24 3FX UK; University of Aberdeen, Aberdeen, UK; Dundee University, Dundee, UK; Stirling University, Stirling, UK; Greenwich University, London, UK; Anglia Ruskin University, Chelmsford, UK

**Keywords:** Demand-control model, Effort-reward imbalance, Occupational stress, Heart rate, Ecological momentary assessment

## Abstract

**Background:**

Stress in health care professionals may reflect both the work and appraisal of work and impacts on the individuals, their patients, colleagues and managers.

**Purpose:**

The purpose of the present study is to examine physiological and psychological effects of stressors (tasks) and theory-based perceptions of work stressors within and between nurses in real time.

**Methods:**

During two work shifts, 100 nurses rated experienced stress, affect, fatigue, theory-based measures of work stress and nursing tasks on electronic diaries every 90 min, whereas heart rate and activity were measured continuously.

**Results:**

Heart rate was associated with both demand and effort. Experienced stress was related to demand, control, effort and reward. Effort and reward interacted as predicted (but only within people). Results were unchanged when allowance was made for work tasks.

**Conclusions:**

Real-time appraisals were more important than actual tasks in predicting both psychological and physiological correlates of stress. At times when effort was high, perceived reward reduced stress.

**Electronic supplementary material:**

The online version of this article (doi:10.1007/s12160-015-9746-8) contains supplementary material, which is available to authorized users.

## Introduction

Although much has been learned about stress, comparatively little work has examined how stressors are experienced and the associated physiological effects in real life. Laboratory studies [1] provide excellent information about the development and recovery of physiological responses to stressors and about individual differences in response to stressors but do not address the real-life stressors that are likely to have lasting health implications. In addition, they typically investigate a single or a small number of stressors such as the mental arithmetic and public speaking involved in the widely used Trier Social Stress Test [2] and cannot simulate the complex pattern of mini-stressors occurring over time in normal life. Real-life studies of major events such as bereavement [3], nuclear accidents [4] or diagnosis of a serious health complaint [5] examine the impact of extreme events over extended periods of time and demonstrate the significant effect of these stressors on mental and physical health but depend on global, retrospective assessments of how serious events were experienced.

Ambulatory psychophysiological studies of stress suggest that the experience of stress or exposure to stressful real-life situations is associated with increases in heart rate and blood pressure (see [6] for a review of this literature). There is also some limited evidence on the determinants of these effects: Zanstra, Johnston & Rasbash [7] found that appraising a real-life stressor (presenting a tutorial paper) as threatening was associated with vascular changes and challenge appraisal had cardiac effects, and Smith, Birmingham & Uchino [8] found that evaluative threat was linked to blood pressure elevations in real life. However, such work is comparatively rare and often limited to a restricted range of real-life stressors. We therefore sought to examine everyday real-life stressors in the workplace as they evolve in real time, to ascertain how individuals evaluated the stressors and to examine how the stressors and the appraisal of the stressors impacted on the individual’s mood and cardiac responses.

It is important to differentiate the objective stressor to which individuals are exposed from the individual’s appraisal or interpretation of the stressor; for example, Lazarus and Folkman [9] differentiated two stages of this individual interpretation process: primary appraisal of the threat, harm or challenge and secondary appraisal of resources to deal with the threat. Individual interpretations are likely to be determined by multiple factors (e.g. personality, coping resources, etc.). In the context of work stress, stressors have been conceptualised in two main theories: the demand-control theory [10] and the effort-reward imbalance theory [11]. The demand-control model posits that the extent to which a work situation is demanding, combined with the degree of control (skill discretion and decision authority) that the person has, determines the strain experienced and the resulting impact on the individual’s experience of stress, job satisfaction and performance. The effort-reward imbalance model proposes that an imbalance between perceptions of effort expended and rewards received determines the degree of experienced stress. These theories have been widely investigated and support found for the hypothesised relationships, especially for the effects of each variable (demand, control, effort, reward) alone, but not always for the hypothesised interactions (demand × control and effort × reward [12]). However, the theories have largely been investigated with questionnaires about past behaviour rather than with measures taken in the real-time context of work where demand, control, effort and reward are actually experienced.

In addition, both theories have mainly been used to explain differences *between* people rather than how stress may fluctuate *within* individuals over time depending on exposure to stressors; i.e. they have investigated *who* experiences stress rather than *when* individuals experience stress. Interventions to reduce stress (by changing either environmental factors to reduce demand or by enhancing individual coping resources [13]) are directed at reducing responses to stressors and depend on the theoretical models being applicable within the individuals. Since it is possible to find support for a theory in between-individual tests but not within individuals and vice versa [14], it is important to establish how well the established work stress theories perform when investigated within individuals. For interventions to be successful, within-person factors must influence the impact of stressors on stress experienced.

Health care providers have been found to have high stress levels [15–17], and stress has been attributed to several organisational factors such as shift working and workload [18]. Nurses’ working day involves a variety of tasks which are potential stressors and which have been found to vary in their perceived stressfulness [19]. Rutledge et al. [19] found that tasks involving direct and indirect care were appraised as more demanding and were associated with greater emotional distress in nurses and physicians but did not test whether workload demand was directly associated with emotional distress. Their study involved ecological momentary assessment (EMA), obtaining responses to randomly timed prompts in the work context. It would therefore have been possible to investigate whether individual fluctuations in workload demand were associated with emotional distress and whether this was true for all individuals. However, the analyses aggregated data over the day, and therefore, the effect of fluctuating demands over the working day was not reported.

Previous studies which have examined demand-control and effort-reward imbalance theories in real-time real-life contexts have found some support for the theories within individuals. A small preliminary study [20] examined 36 nurses who completed hand-held computer diary ratings every 90 min over three work shifts and found that the ratio of demand to control and of effort to reward was related to responses on a single visual analogue scale of experienced stress, supporting both theories. A larger study with more comprehensive assessments [21] established that negative affect was predicted by high demand/effort, low control and low reward. Control and reward moderated the effects of demand/effort. Although it was concluded that perceptions of work have influence in the same way within and between individuals, this was not tested directly.

As outlined above, the effects of work stress may be due either to the appraisals of the tasks undertaken or to the tasks themselves: for example, nurses may feel tense because they are delivering direct patient care or because they perceive this task to be demanding, effortful, unrewarding, etc. Whereas Johnston et al. [21] assessed the appraisal of work stressors, they did not assess the tasks being undertaken. It is therefore possible that the effects on mood were due either to the nature of the work stressors, including the tasks being undertaken, or to the individuals’ evaluation of the work as demanding, effortful, controllable and rewarding. The implications for the design of interventions to reduce the negative effects of work stress would be quite different if the stress was due to the tasks undertaken than if it was due to cognitions about the work: the first interpretation would require the work to be changed, whereas the latter would require cognitive restructuring and may be more easily accommodated alongside the need to deliver nursing care.

A final limitation of earlier studies is that they only examined the subjective experience of stress and did not investigate effects on physiological functioning. The advantages of investigating physiological functioning are considerable: such measures avoid confounding by response bias inherent in subjective ratings, and stress ratings can be related to concrete physiological outcomes that may have direct implications for the individuals’ health [22–24]. We therefore sought to investigate the relationship between objective stressors (nursing tasks), appraisals of stressors conceptualised as the theoretical determinants of work stress (demand, control, effort and reward), experienced stress (mood ratings) and cardiac response (heart rate) using EMA methods and with multilevel statistical analyses to identify both between-person and within-person relationships. We additionally assessed positive mood and fatigue.

### Research Questions

1. Are heart rate and experienced stress predicted by appraisal of work stressors as proposed by the demand-control and effort-reward imbalance theories of work stress: (a) between nurses and (b) within individual nurses over time?
2. Are observed predictions of heart rate and experienced stress from appraisal of work stressors explained by *perceptions* of work tasks over and above the objective nature of these tasks?

## Methods

A full protocol for the study has been published previously [25]. In this paper, essential methodological information is summarised. The study was approved by NHS committee NRES Committees-North of Scotland (Reference 10/S0801/87; date 11 January 2011).

### Design and Procedure

Nurses were recruited from advertisements posted on medical and surgical wards in a large teaching hospital in the North East of Scotland. They completed initial questionnaires and wore a heart rate and activity monitor for the duration of their participation in the study. They completed EMA items using electronic diaries every 90 min over two 12-h working shifts.

### Participants and Recruitment

All qualified nurses on medical and surgical wards with more than 20 beds were eligible. Nurses who expressed an interest received an information pack, and those who chose to participate returned a signed consent form on receipt of which a member of the research team made arrangements for data collection. Nurses received a £25 voucher for a major store on completing testing.

### Measures

#### *Physiological Recording (Monitors Were Worn Continuously Throughout Two Shifts)*

Heart rate, activity and energy expenditure were obtained from Actiheart monitors (Cambridge Neurotechnology, Cambridge, UK) using the procedures specified by the manufacturers, CamNTech. The standard Actiheart routines [26] were used to edit out artefactual beats, The Actiheart software operates on 15-s epochs. Artefacts are detected in a multistage process. First individual beats of exactly 2000 ms (the longest inter-beat interval stored and indicating a missed beat) are rejected, as are beats that differ from the preceding beat by more than 20 times the average difference in the epoch. The last 16 good inter-beat intervals are then averaged, and any that are outside ±25 % of this average are removed and the remaining inter-beat intervals re-averaged and converted to beats per minute. In addition, all 1-min means of below 40 bpm or above 170 were excluded. The data were of high quality, and only 0.01 % of 1-min means were rejected.

#### *PDA Electronic Diary Measures (of Mood, Appraisals of Work Stressors and Nursing Work Tasks Completed, on Average, Every 90 Min over Two Shifts)*

[Full details of the measures with screen grabs of the personal digital assistant (PDA) are available in Appendix 1: supplementary material 1.]

The EMA measures were delivered on PDAs (Dell Axim 51, Round Rock, TX, USA) using the Pocket Interview software [27]. Data entry was prompted by an audible alarm that sounded for 8 s approximately every 90 min during the shift (with a window of ±15 min determined randomly). If a participant did not respond within 2 min, the alarm sounded again and this continued for a maximum of 10 cycles. If the participant did not respond, then the device closed down until next scheduled alarm occurred. The participant could postpone (‘snooze’) an alarm for up to 60 min if it occurred at a time when it was not possible to make a diary entry.

#### *Mood/Experienced Stress*

Twelve items from the University of Wales Institute of Science and Technology (UWIST) mood scale [28] that loaded substantially and specifically on each relevant dimension measured the following: experienced stress (‘tense-arousal’), affect (‘hedonic tone’) and ‘fatigue’ (the inverse of what Mathews et al. [28] call ‘energetic arousal’). Participants rated their mood ‘now’ on visual analogue scales labelled ‘no’ to ‘yes’ which were scored from 0 to 100. High scores indicate high experienced stress, positive affect and high fatigue. The between- and within-person reliabilities, which are akin to Cronbach alphas, were calculated using established procedures [29]. The between-person reliability was calculated as the average over the two shifts. The reliabilities in this study were as follows: affect: between 0.98, within 0.6; experienced stress: between 0.97, within 0.63; and fatigue: between 0.98, within 0.65, which were satisfactory.

#### *Appraisals of Work Stressors: Theoretical Determinants of Work-Related Stress*

The main determinants of work-related stress from the Karasek [9] demand-control model and the Siegrist [10] effort-reward imbalance model were assessed for the previous 10 min using four analogue scales (demand, control, effort, reward) and binary items based on standard questionnaire measures [30, 31]. The different types of scales were used to reduce common method variance. The binary items were five demand items (work fast, work hard, do too much, interrupted and enough time available), three control items (requiring a high level of skill, allowed a lot of say in what they did, allowed to make the main decisions about what they did), four effort items (under constant pressure, had a lot of responsibility, under a lot of physical demand and interrupted), and three reward items (being valued, appreciated, respected). The analogue items were scored from 0 to 1 to give them comparable weight to the binary items. The reliabilities were as follows: demand: between 0.96, within 0.77; control: between 0.88, within 0.45; effort: between 0.92, within 0.69; and reward: between 0.94, within 0.85. Control, within person, was not reliable. Examination of the inter-item correlations indicated that the item ‘requiring a high level of skill’ was negatively correlated with the other control items between people and uncorrelated with the other control items within people. When this item was dropped, the within-person reliability was slightly improved to 0.50 (between remained high at 0.95), and so the skill item was removed from the scale.

#### *Nursing Work Tasks*

The categorisation of nursing tasks was based on the Work Observation Method by Activity Timing (WOMBAT, [32]) classification system. The WOMBAT classification results in high inter-rater reliability of classifications, and very few tasks are unclassified. Nursing tasks were divided into ten categories (direct patient care, indirect care, medication, documentation, professional communication, in transit, social/break, supervision, ward related and other). Participants classified their main work task for the preceding 10 min during each diary entry.

Prior to beginning the first participation shift, nurses were trained to use the WOMBAT work task classification scheme using a specially prepared errorless training booklet (see Appendix 2: supplementary materials 2) and were tested on this prior to their first shift. All participants coded the test material correctly, indicating that the tasks were classified correctly with no variation between individuals. Forty-four participants did not complete the pretraining in their own time and, instead, went through the training booklet with the researcher prior to the first shift.

### Procedure

Consenting nurses were sent the nursing task classification pretraining booklet and completed the assessment prior to the first participation shift. A member of the research team met the nurse on the ward before the start of shift, and nurses were equipped with the Actiheart device using Ambu Blue Sensor-R electrodes, following the procedures recommended by CamNTech. Nurses were given a preprogrammed PDA, completed the rest of their shift as usual and were met by the researcher at the shift end for equipment retrieval.

### Analyses

EMA diaries plus heart rate data were obtained from all 100 nurses, over 196 shifts. The sample was comparable to the total workforce on the wards tested on age and years since qualifying but contained slightly more men (7 out of 100) compared to the 3.5 % of the population sample (see Table 1 for details). Seventy-five percent of participation shifts were day shifts, and 4 % [8] were at the weekend.

**Table 1:** Participants: demographic, professional and participation information

	Medical wards	Surgical wards	Comparison group^a^
*N*	47	53	425
Gender (% female)	93.6 %	92.5 %	96.5 %
Age in years (mean, SD)	35.9 (9.5)	36.9 (10.2)	36.9 (10.3)
Body mass index (mean, SD)	27.8 (6.1)	25.9 (5.1)	
Qualifications (% graduate level)	47.9 %	34.0 %	
Pay grade (% in lower bands)	69.6 %	76.9 %	
Years registered as a nurse (mean, SD)	9.4 (8.7)	11.5 (9.9)	11.0 (9.5)
Years working on ward (mean, SD)	5.0 (4.9)	5.4 (5.0)	
Number of wards included	7	7	14
Number of shifts included	92	104	

^a^All nurses employed on participating wards

The work stress models were tested both between and within participants in the same analyses. The demand-control and effort-reward imbalance models were examined in analyses with four main effects (demand or effort and control or reward) averaged within people and grand mean centred to assess between-person effects and the individual assessments, centred within individuals, to determine within-person effects. Interaction terms representing demand × control and effort × reward were calculated for both between and within effects.

The self-rated diary data were analysed using a three-level multilevel model with repeated measures nested within shifts and nested within participants. The intercept was always treated as a random effect at all levels. The regression slopes of other dependent variables were all treated as fixed. Shift (first or second) and time into the shift (in h) were included in the analysis to reduce extraneous variance. Heart rate was analysed as a four-level multilevel model. The ten 1-min means preceding each diary entry (the diary assessments covered a 10-min period) were treated as the lowest-level variable which was then nested within occasions, shifts and finally participants. The ten separate minute periods were fitted by a quadratic polynomial since heart rate decreased about the time that the diary entry was made. Activity derived from the chest-mounted accelerometer in the Actiheart was included in the analysis to allow for the effects of activity on heart rate. It was log transformed to reduce positive skew. Most of the repeatedly measured data were moderately autocorrelated, and a multilevel model including autocorrelation between occasion level residuals was used [33]. The analyses were carried out using MLwiN v2.30. The alpha level was set at *p* < 0.05 with Bonferroni correction for the six effects examined in each analysis. These analyses were repeated allowing for the effects of each of the nursing tasks.

## Results

### Diary Completion Rate

Overall, 1453 (98.5 %) out of a possible 1476 diary entries were completed. The diary was opened with median delay of 30 s, and the modal snooze time was 10 min. Three hundred and fifty-nine (25 %) entries were snoozed.

*Summary data* are shown in Table 2. On average, participants were not very stressed nor fatigued and were in a positive mood. They rated the work as moderately demanding and effortful, and they felt that they had considerable control and were fairly highly rewarded. The intraclass correlations show that the mood measures varied substantially between people, whereas for the work stressor appraisal measures, the intraclass correlations were low with the exception of reward, indicating that they largely varied within people over time. The intercorrelations were usually higher between people than within.

**Table 2:**
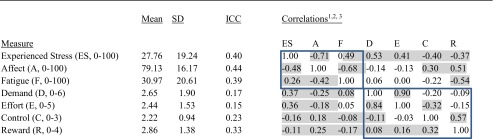
Means, standard deviations (between and within participants), intraclass correlations (ICC) and correlations for the EMA measures (between and within participants)

^a^Correlations shown above the diagonal (i.e. top right of matrix) are between-participant correlations, and those below the diagonal (i.e. bottom left of matrix) are within-participant correlations

^b^Shaded figures indicate significant correlations

^c^Boxes contain correlations between experienced stress, affect and fatigue and between work stress measures

### Is Heart Rate Predicted by Appraisal of Work Stressors as Proposed by the Demand-Control and Effort-Reward Imbalance Theories of Work Stress—Between Individual Nurses? Within Individual Nurses over Time?

Table 3 shows the results for heart rate adjusted for activity. Between-person results show that heart rate was higher in those who appraised their work as more demanding or requiring more effort with an increase of 1 standard deviation in demand being associated with an increase of almost 3 bpm in heart rate. Within-person results show that adjusted heart rate was also higher during periods of high demand by slightly more than 1 bpm for 1 SD increase in demand. Most nurses’ heart rate increased as demand increased; not a single nurse showed the opposite pattern. Effort was related to heart rate similarly to demand. Control and reward were unrelated to heart rate.

**Table 3:** Demand-control and effort-reward imbalance model for physiological measures: estimated beta weights (standard error) for fixed effects and variances for random effects with and without allowing for work tasks

		Demand-control model			Effort-reward imbalance model	
		Heart rate	Heart rate allowing for work tasks		Heart rate	Heart rate allowing for work tasks
Fixed effects						
	Intercept	83.86 (1.19)	84.26 (1.21		83.80 (1.16)	84.71 (1.18)
	Shift (1 or 2)	−0.10 (0.59)	−0.17 (0.58)		−0.16 (0.61)	−0.23 (0.59)
	Time into shift (h)	−0.31 (0.05)	−0.27 (0.05)		−0.30 (0.05)	−0.26 (0.05)
	Linear effect over period before diary entry.	−0.46 (0.07)	−0.46 (0.07)		−0.46 (0.07)	−0.46 (0.07)
	Quadratic effect over period before diary entry	−0.03 (0.01)	−0.03 (0.01)		−0.03 (0.01)	−0.03 (0.01)
	Accelerometer measure of activity	1.69 (0.02)	1.69 (−0.020)		1.69 (0.02)	1.69 (0.02)
Between persons	Demand	2.86* (1.11)	2.89* (1.11)	Effort	3.25* (1.40)	3.26* (1.40)
	Control	2.32 (2.11)	2.29 (2.11)	Reward	−0.09 (1.24)	−0.16 (1.24)
	Demand × control	0.08 (2.15)	0.17 (2.15)	Effort × reward	0.54 (1.50)	0.57 (1.50)
Within persons over time	Demand	0.84* (0.11)	0.58* (0.13)	Effort	0.86* (0.14)	0.54* (0.15)
	Control	0.34 (0.23)	0.32 (0.23)	Reward	0.0 (0.17)	−0.08 (0.18)
	Demand × control	1.01 (0.13)	0.07 (0.13)	Effort × reward	0.20 (0.12)	0.23 (0.12)
Random effects						
	Person	104.76 (16.07)			106.29 (16.34)	
	Shift	11.04 (2.40)			11.68 (2.50)	
	Time into shift	31.00 (1.57)			31.35 (1.58)	
	Period before diary entry	30.97 (0.50)			30.96 (0.50)	
	Alpha (autocorrelation)	13.34 (0.44)			13.32 (0.44)	

Alpha is a time series parameter: the covariance between two observations *t* time units apart is alpha × 1 / *t*

* *p* < .05 Bonferroni corrected

### Are Experienced Stress, Affect and Fatigue Predicted by Appraisal of Work Stressors as Proposed by the Demand-Control and Effort-Reward Imbalance Theories of Work Stress—Between Individual Nurses? Within Individual Nurses over Time?

**Fig. 1:**
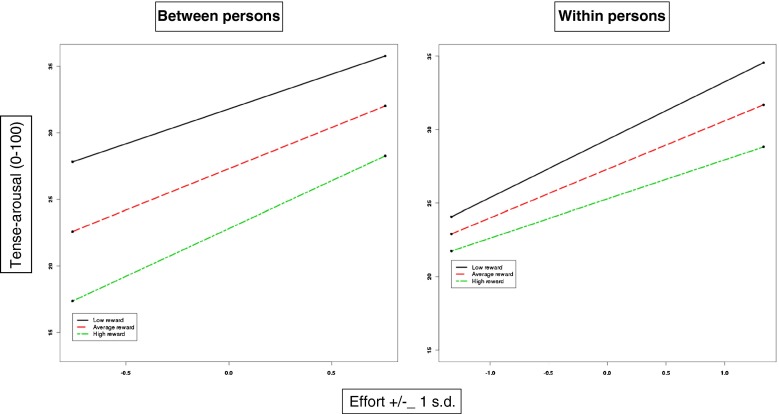
Comparison of between- and within-person results for ERI model predicting tense arousal (experienced stress)

In Table 4, it can be seen that demand and control predicted experienced stress in a similar way both between and within participants. Experienced stress was higher when demand was, on average, higher or when it was higher at a particular moment, and experienced stress was lower when control was higher. Demand and control did not interact. Affect was predicted by control across participants; nurses who, on average, reported higher control had more positive affect. Average level of demand was not related to affect nor was the interaction significant. Within participants, the main effects were significant, and in the expected direction, i.e. at times when demand was high, affect was lower; moments of higher control were associated with more positive affect; however, although the interaction of demand and control was in the predicted direction, it was not significant. Fatigue only related to control, both between and within, high control being associated with less fatigue. The effort-reward model was tested in the same way as demand-control (see Table 5). The main effects of effort and reward on experienced stress were significant between participants, and all within-participant effects, including the interaction, were significant (see Fig. 1). Only reward related to affect between participants. However, all the within-participant effects, including the interaction, were significant and in the predicted direction. Fatigue related to reward both within and between participants.

**Table 4:** Demand-control model for experienced stress, affect and fatigue: estimated beta weights (standard error) for fixed effects and variances for random effects

		Experienced stress	Affect	Fatigue
Fixed effects				
	Intercept	27.48 (1.41)	81.75 (1.41)	20.30 (1.72)
	Shift (1 or 2)	−0.94 (0.97)	−2.33 (1.10)	0.12 (1.19)
	Time into shift (h)	0.28 (0.12)	−0.34 (0.09)	2.05 (0.13)
Between persons	Demand	5.23* (1.17)	−0.49 (1.21)	−0.07 (1.46)
	Control	−6.99* (2.21)	6.56* (2.30)	−6.30* (2.77)
	Demand × control	3.89 (2.30)	−3.23 (2.39)	3.40 (2.88)
Within persons over time	Demand	2.77* (0.22)	−1.10* (0.16)	0.26 (0.20)
	Control	−2.02* (0.48)	1.83* (0.31)	−1.18* (0.39)
	Demand × control	−0.43 (0.25)	0.41 (0.18)	0.00 (0.22)
Random effects				
	Person	100.52 (17.86)	104.60 (19.50)	159.83 (27.65)
	Shift	1.87 (7.95)	27.90 (8.65)	0 (0)
	Time into shift	181.44 (9.19)	98.79 (5.21)	193.44 (9.30)
	Alpha (autocorrelation)	41.35 (8.79)	35.36 (4.77)	94.33 (7.96)

Alpha is a time series parameter: the covariance between two observations *t* time units apart is alpha × 1 / *t*

* *p* < .05 Bonferroni corrected

**Table 5:** Effort-reward imbalance model for experienced stress, affect and fatigue: estimated beta weights (standard error) for fixed effects and variances for random effects

		Experienced stress	Affect	Fatigue
Fixed effects				
	Intercept	27.29 (1.41)	81.81 (1.26)	19.93 (1.51)
	Shift (1 or 2)	−1.11 (0.97)	−2.26 (1.05)	0.13 (1.17)
	Time into shift (h)	0.27 (0.12)	−0.34 (0.09)	2.07 (0.13)
Between persons	Effort	6.16* (1.52)	−1.16 (1.36)	−0.47 (1.56)
	Reward	−5.06* (1.33)	6.85* (1.19)	−8.59* (1.39)
	Effort × reward	1.09 (1.61)	−2.04 (1.45)	3.08 (1.69)
Within persons over time	Effort	3.30* (0.27)	−1.20* (0.19)	−0.22 (0.24)
	Reward	−1.92* (0.34)	2.15* (0.24)	−1.51* (0.30)
	Effort × reward	−0.61* (0.24)	0.84* (0.17)	−0.04 (0.21)
Random effects				
	Person	111.21 (19.13)	80.66 (15.74)	112.72 (20.80)
	Shift	0 (0)	22.99 (7.95)	0 (0)
	Time into shift	185.92 (8.92)	98.11 (5.17)	189.37 (9.12)
	Alpha (autocorrelation)	43.83 (7.25)	35.42 (4.73)	90.71 (7.82)

Alpha is a time series parameter: the covariance between two observations *t* time units apart is alpha × 1 / *t*

* *p* < .05 Bonferroni corrected

### Are Observed Predictions of Heart Rate and Experienced Stress from Appraisal of Work Stressors Explained by Perceptions of Work Tasks over and Above the Objective Nature of These Tasks?

The work stress models were tested allowing for the effects of tasks. This was done by including the tasks as categorical variables in the analyses. Direct patient care, the largest single category, was used as the reference category. The results for the effects of the main dependent variables on heart rate are shown in Table 3. There was some attenuation of the relationships, but all significant effects remained so. All the significant effects of the theoretical determinants of work stress on experienced stress, affect and fatigue were little affected (Table 6). The between-participant effects were not affected, and all effects that were previously significant were essentially unchanged. Tables 3 and 6 have been simplified by excluding the task categories and (in Table 6) the random effects; the complete tables are included in Appendix 3: supplementary material 3.

**Table 6:** Work stress model results for appraisal of stress, affect and fatigue allowing for work tasks: estimated beta weights (standard error) for fixed effects

		Experienced stress	Affect	Fatigue
Demand-control model				
Between	Demand	5.21* (1.16)	−0.49 (1.21)	−0.05 (1.46)
	Control	−6.93* (2.20)	6.40* (2.29)	−6.08* (2.76)
	Demand × control	4.13 (2.29)	−3.29 (2.38)	3.38 (2.89)
Within	Demand	2.75* (0.25)	−1.19* (0.18)	0.50 (0.23)
	Control	−2.03* (0.45)	1.81* (0.32)	−1.08* (0.40
	Demand × control	−0.45 (0.25)	0.43 (0.18)	−0.02 (0.22)
Effort-reward imbalance model				
Between	Effort	6.09* (1.52)	−1.10 (1.36)	−0.55 (1.58)
	Reward	−5.13* (1.33)	−6.86* (1.19)	−8.56* (1.39)
	Effort × reward	1.14 (1.61)	−2.07 (1.44)	3.06 (1.68)
Within	Effort	3.20* (0.31)	−1.07* (0.22)	0.20 (0.28)
	Reward	−1.98* (0.35)	2.17* (0.25)	−1.48* (0.31)
	Effort × reward	−0.59* (0.24)	0.81* (0.17)	−0.03 (0.21)

* *p* < .05 Bonferroni corrected

## Discussion

This study assessed stress in a real-life work situation, collecting information in real time over two work shifts, on stressors (work tasks), appraisals of the stressors (theoretical determinants), experienced stress (rated tense-arousal), fatigue, positive mood and cardiac response. It has succeeded in collecting a large data set with few missing observations. It is therefore possible to examine the value of the two theoretical models demand-control and effort-reward Imbalance in accounting for both psychological and physiological states, making allowance for activity levels. Some support was found for the models for psychological and physiological responses and in predicting differences both between individuals and predicting changes within people over time. The predictive value of the models could not simply be attributed to a confounding of the stressor and the appraisal of that stressor, as the findings were virtually unchanged when allowance was made for specific work tasks. However, there were also aspects of each model which were not supported and there was some evidence of different predictions within and between people.

Considering first the demand-control model, demand predicted increases in heart rate and in experienced stress, but not fatigue. This was found both between and within participants and when allowance was made for the objective stressors conceptualised as the tasks that the nurses were engaged in. Demand predicted reduced positive affect within participants but not between. By contrast, control did not predict cardiac response but was predictive of feeling less stressed and more positive both between and within respondents. The interaction between demand and control was not significant for heart rate, experienced stress, affect nor fatigue. These results parallel the finding of Kamarck et al. [34] that demand related to systolic and diastolic blood pressure but fail to support their finding that control also predicted the cardiovascular response.

The results showing a consistent effect of demand and control but not the interaction are similar to those found in many previous studies [12]. Thus, there appears to be a consensus about the effects of demand and control, but further work is necessary to clarify when an interaction between demand and control is likely to occur. Häusser et al. [12] suggest a matching principle, essentially that the type of control studied has to be relevant in order to buffer the effects of demand; for example, if demand is about time-pressure and control allows control of scheduling, then control would be likely to buffer demand. If, however, the type of control available does not align with the type of demand experienced, the hypothesised interaction would be less likely to occur. Häusser et al. [12] get some support for this position from their review. They note that de Jonge & Dorman [35] extend matching to the response so if the strain measured is emotional, then they consider that the control and demand should be also; for example, if the stressor is a demanding and highly controlling manager, then the impact should be on emotional strain; in contrast, the effects of having extremely physically dependent patients might be on physical measures of response. Support has been obtained for what de Jonge terms the triple match principle [36]. There appears little doubt that, as Bakker has argued for some time [13], demand and resource, conceived quite widely to include not only control but also reward, social support and other personal and environmental factors, contribute to occupational stress and its amelioration.

Previous work has been dominated by evidence of effects between people. These results confirm that demand and control not only predict who will be stressed but also additionally predict when individuals will be stressed. The demand effects cannot simply be attributed to the extra physiological requirements of demanding tasks as the heart rate analyses allowed for activity levels and therefore allow one to conclude that psychological demand is having a physiological impact over and above that associated with the physical demands of a task. Although a small effect, there were no individuals in this sample of 100 for whom increased psychological demand reduced heart rate. Given the regular and frequent repetition of this increased cardiac response every work day, this might have a cumulative, longer-term impact on health. Unlike demand, increased control over work not only resulted in lower experienced stress, but also impacted on other moods, increasing positive affect and decreasing fatigue but did not predict heart rate.

The effort-reward imbalance model had similar results between persons to those found for the demand-control model, i.e. an effect of effort on heart rate and experienced stress; an effect of reward on experienced stress, affect and fatigue; and no interaction effects. However, the within-person results are different: the interaction is significant for both experienced stress and affect, and in each case, the positive effect of reward increased as effort increased, as predicted by theory. This may reflect the better within-person reliability of the measure of reward compared to control. The predictive value of the theoretical models was virtually unchanged when allowance was made for the tasks being undertaken.

Some nursing tasks are seen to be more stressful than others [18], and in this study, the reported tasks were used as an index of varying stressors. Nurses reported more experienced stress during episodes of direct care than in other activities, and this was significantly different from periods when documentation was the main task [37]. Nevertheless, the findings for the appraisals of stress continued to have similar size and significance of effects with and without allowance for work tasks, suggesting that the appraisals of work may have an impact which is quite independent of the actual work and the stressors involved. Importantly, these results would suggest that interventions might be effective in reducing both the physiological and psychological aspects of stress without needing to change work tasks. In the context of nursing and health care, this makes practical sense as it would be impossible to remove tasks involving direct patient care, tasks which nurses additionally reported to be rewarding [37].

Findings for other states are different from those for experienced stress. Demand and effort had no effect on fatigue. This is surprising as one might expect effort to increase fatigue levels both due to physical exertion and due to the demands of sustained cognitive effort. There is ample evidence that following times with high cognitive or motivational demands, people lack the resources for self-control and the ability to engage effectively in further tasks, a process labelled ‘ego depletion’ [38]. It may be that in the context of a long working day, demand has both a tiring and an energising effect. There was no relationship between average demand and affect; i.e. nurses who generally saw their work as less demanding were no happier. However, there was a within-person effect: people were less happy at times when demand was high. Fatigue was decreased, and positive affect increased in persons who felt they had control and, at times, when individuals felt themselves to be in control. Similarly, affect and fatigue both showed positive effects of reward between and within persons.

Although many of the findings are similar in explaining differences between people and within individuals over time, there are some differences. It is therefore important to emphasise the need to test models within persons before suggesting that the theory offers intervention possibilities. Between-person findings do not offer a basis for intervention. Based on the current findings, one might propose that interventions should aim to reduce the demand and increase control of work and that this might be achieved by cognitive methods such as cognitive restructuring, without changing the nature of the tasks undertaken. The small intra-class correlations for demand, effort and control suggest that there is greater variation within than between people supporting the aim of changing cognitions occurring at times of high demand or low control. Results from a recent review and meta-analysis provide support: cognitive, behavioural and mindfulness-based approaches were effective in reducing stress in practicing physicians and in medical students [39]. Additionally, attention might be paid to increasing the nurse’s feeling of being appreciated or otherwise rewarded. This might be achieved by changes at either an organisational or personal level.

## Limitations

EMA studies are burdensome, and there may be bias in the individuals who consent to take part. We included 100 from a possible pool of 425 nurses. Additional nurses were willing to participate, but we ceased recruitment when sufficient numbers were achieved and so cannot report a true ‘consent rate’. The data in Table 1 suggest that the recruited nurses are similar to the total group in terms of the variables assessed. Nevertheless, they might still be biased on factors relevant to the study. Nurses who were particularly stressed or critical of the work situation might have participated in order to have their views acknowledged, or alternatively, the most stressed nurses might have avoided adding our study to their stressors or indeed might have been on sick leave. Such biases might be important in a study of prevalence of stress but are less important for investigation of relationships between theoretical constructs related to stress. If anything, the omission of nurses with more extreme high or low stress would be likely to diminish the between-person effects found. Biases in the sample would be even less likely to have an effect on relationships over time within persons.

In all of the analyses, the term prediction is used to indicate statistical rather than temporal prediction. Whereas reports of stressors (tasks) and appraisal of these stressors refer to the previous 10 min, these reports were obtained at the same time as the other diary entries. In addition, although the theories investigated propose causal relationships, this study only tests correlation. Where results support the theories, they can be seen as supportive but not testing the causal hypotheses. However, where results do not support the theories, this is challenging to the theory as the causal relationships proposed should result in evidence of correlation.

The WOMBAT classification of work tasks was used as an objective index of the stressor but was reported by individuals at the same time as they appraised the stressor. These appraisals may therefore have influenced the classifications. However, evidence from previous work [28] and from the classification of tasks in training for the current study indicates that the WOMBAT categories produce reliable classification, with individuals agreeing in their classification of tasks. Some tasks may be inherently more stressful than others, and within WOMBAT categories, there is likely to be variation. Nevertheless, the appraisal of the tasks by the nurses is associated with psychological and physiological changes above and beyond that due to the tasks per se.

## Conclusions

This study is exceptional in investigating theoretical models of stress both within and between people in real-life nursing and in real time over working shifts. The results show support for the effects of demand and effort on both cardiac responses and on psychological measures of experienced stress. Perceptions of control over work were associated with more positive moods, i.e. less tense, more cheerful and less fatigue. However, neither demand-control nor effort-reward imbalance models received full support as there was limited evidence of the hypothesised interactions between demand and control or between effort and reward.

These results were found both between nurses and within individual nurses over two work shifts. However, between and within analyses did not always produce the same findings, highlighting the inappropriateness of generalising from between-person studies to within-person conclusions. Within-person findings are of particular value in that they may more appropriately be used as a basis for designing interventions. Allowing for the work tasks as an index of variation in stressors did not affect any of the results, suggesting that interventions to reduce stress might appropriately use cognitive methods to target perceptions without the need to alter the actual work.

## Electronic supplementary material

Below is the link to the electronic supplementary material.

Appendix 1 Supplementary Material on PDA items to accompany: “Stressors, appraisal of stressors, experienced stress and cardiac response: a real-time, real-life investigation of work stress in nurses.” (DOCX 211 kb)

Appendix 2 Supplementary material – WOMBAT training manual to accompany: “Stressors, appraisal of stressors, experienced stress and cardiac response: a real-time, real-life investigation of work stress in nurses.” (DOC 6.02 mb)

Appendix 3 Supplementary Material on effects of work tasks to accompany: “Stressors, appraisal of stressors, experienced stress and cardiac response: a real-time, real-life investigation of work stress in nurses.” Complete versions of Tables 3 and Table 6 in the paper showing the estimated beta weights and SE for the work tasks. In all cases direct patient care was used as the reference category (DOCX 17.8 kb)

## References

[1] Chida Y, Hamer M (2008). Chronic psychosocial factors and acute physiological responses to laboratory-induced stress in healthy populations: A quantitative review of 30 years of investigations. Psychol Bull.

[2] Kirschbaum C, Pirke KM, Hellhammer DH (1993). The ‘trier social stress test’—a tool for investigating psychobiological stress responses in a laboratory setting. Neuropsychobiology.

[3] Bodnar JC, Kiecolt-Glaser JK (1994). Caregiver depression after bereavement: Chronic stress isn’t over when it’s over. Psychol Aging.

[4] Baum A, Gatchel RJ, Schaeffer MA (1983). Emotional, behavioral, and physiological effects of chronic stress at Three Mile Island. J Consult Clin Psychol.

[5] Thompson SC, Nanni C, Levine A (1996). The stressors and stress of being HIV-positive. AIDS Care.

[6] Zanstra YJ, Johnston DW (2011). Cardiovascular reactivity in real life settings: Measurement, mechanisms and meaning. Biol Psychol.

[7] Zanstra YJ, Johnston DW, Rasbash J (2010). Appraisal predicts hemodynamic reactivity in a naturalistic stressor. Int J Psychophysiol.

[8] Smith TW, Birmingham W, Uchino BN (2012). Evaluative threat and ambulatory blood pressure: Cardiovascular effects of social stress in daily experience. Health Psychol.

[9] Lazarus RS, Folkman S. *Stress, appraisal, and coping.* New York, Springer Publishing Company: 1984

[10] Karasek RA Jr. Job demands, job decision latitude, and mental strain: Implications for job redesign. *Adm Sci Q*. 1979; 285–308.

[11] Siegrist J (1996). Adverse health effects of high-effort/low-reward conditions. J Occup Health Psychol.

[12] Häusser JA, Mojzisch A, Niesel M, Schulz-Hardt S (2010). Ten years on: A review of recent research on the job demand–control (−support) model and psychological well-being. Work Stress.

[13] Bakker AB, Demerouti E, Sanz-Vergel AI (2014). Burnout and work engagement: The JD–R approach. Annu Rev Organ Psychol Organ Behav.

[14] Johnston DW, Johnston M (2013). Useful theories should apply to individuals. Br J Health Psychol.

[15] Mann S, Cowburn J (2005). Emotional labour and stress within mental health nursing. J Psychiatr Ment Health Nurs.

[16] Williams S, Michie S, Pattani S. *Improving the health of the NHS workforce: Report of the partnership on the health of the NHS workforce.* Nuffield Trust; 1998.

[17] Jones MC, Johnston DW (1997). Distress, stress and coping in first-year student nurses. J Adv Nurs.

[18] McVicar A (2003). Workplace stress in nursing: A literature review. J Adv Nurs.

[19] Rutledge T, Stucky E, Dollarhide A (2009). A real-time assessment of work stress in physicians and nurses. Health Psychol.

[20] Johnston DW, Beedie A, Jones MC (2006). Using computerized ambulatory diaries for the assessment of job characteristics and work-related stress in nurses. Work Stress.

[21] Johnston DW, Jones MC, Charles K, McCann SK, McKee L (2013). Stress in nurses: Stress-related affect and its determinants examined over the nursing day. Ann Behav Med.

[22] McEwen BS (1998). Stress, adaptation, and disease: Allostasis and allostatic load. Ann N Y Acad Sci.

[23] Schnall PL, Schwartz JE, Landsbergis PA, Warren K, Pickering TG (1992). Relation between job strain, alcohol, and ambulatory blood pressure. Hypertension.

[24] Bosma H, Marmot MG, Hemingway H, Nicholson AC, Brunner E, Stansfeld SA (1997). Low job control and risk of coronary heart disease in Whitehall II (prospective cohort) study. BMJ.

[25] Farquharson B, Bell C, Johnston D (2013). Nursing stress and patient care: Real-time investigation of the effect of nursing tasks and demands on psychological stress, physiological stress, and job performance: Study protocol. J Adv Nurs.

[26] CamNTech *The Actiheart user’s manual V4.0.109.* 2013.

[27] Morrison K, Ricketts IW, Jones MC, Johnston DW, Pitts NB, Sullivan FM. Pocket interview: A secure electronic data collection and diary tool. *Ehealth International*. 2009; 1–9.

[28] Matthews G, Jones DM, Chamberlain AG (1990). Refining the measurement of mood: The UWIST mood adjective checklist. Br J Psychol.

[29] Cranford JA, Shrout PE, Iida M, Rafaeli E, Yip T, Bolger N (2006). A procedure for evaluating sensitivity to within-person change: Can mood measures in diary studies detect change reliably?. Pers Soc Psychol Bull.

[30] Karasek R, Brisson C, Kawakami N, Houtman I, Bongers P, Amick B (1998). The job content questionnaire (JCQ): An instrument for internationally comparative assessments of psychosocial job characteristics. J Occup Health Psychol.

[31] Siegrist J, Starke D, Chandola T (2004). The measurement of effort–reward imbalance at work: European comparisons. Soc Sci Med.

[32] Westbrook JI, Ampt A (2009). Design, application and testing of the work observation method by activity timing (WOMBAT) to measure clinicians’ patterns of work and communication. Int J Med Inf.

[33] Rasbash J, Steele F, Browne W, Goldstein H (2013). A user’s guide to MLwiN.

[34] Kamarck TW, Janicki DL, Shiffman S (2002). Psychosocial demands and ambulatory blood pressure: A field assessment approach. Physiol Behav.

[35] De Jonge J, Dormann C (2006). Stressors, resources, and strain at work: A longitudinal test of the triple-match principle. J Appl Psychol.

[36] Van de Ven B, Jonge J, Vlerick P (2014). Testing the triple-match principle in the technology sector: A two-wave longitudinal panel study. Appl Psychol.

[37] Johnston, M., Johnston, D., Allan, J., Farquharson, B., Jones, M., Ricketts, I., Schofield, P. & Bell, C. Nursing stress and patient care: Real-time investigation of the effect of nursing tasks and demands on psychological and physiological stress and job performance. Final Report to Chief Scientist Office of grant CZH 4/460. 2013. 10.1111/jan.12090 23387943

[38] Baumeister RF, Bratslavsky E, Muraven M, Tice DM (1998). Ego depletion: Is the active self a limited resource?. J Pers Soc Psychol.

[39] Regehr C, Glancy D, Pitts A, LeBlanc VR (2014). Interventions to reduce the consequences of stress in physicians: A review and meta-analysis. J Nerv Ment Dis.

