# Supplementary material for: Stressors, Appraisal of Stressors, Experienced Stress and Cardiac Response: A Real-Time, Real-Life Investigation of Work Stress in Nurses
Source: Ann Behav Med. 2015 Nov 25;50:187–97. doi: 10.1007/s12160-015-9746-8 (PMC4823345; doi:10.1007/s12160-015-9746-8)

Appendix 1. Supplementary Material on PDA items to accompany:

“Stressors, appraisal of stressors, experienced stress and cardiac response: a real-time, real-life investigation of work stress in nurses.”

**Mood PDA items**

Items were scored from 0-100 and anchored No-Yes. Adjectives were selected from the UWIST Mood Adjective Checklist (Matthews, Jones & Chamberlain, 1990) to measure experienced stress (ES) affect (A), and fatigue (F))

**Analogue scales (No-Yes, 0-100):**

“How are you feeling?”

Alert (F)

Tired (F)

Happy (A)

Stressed (ES)

Angry (A)

Energetic (F)

Sad (A)

Nervous (ES)

Cheerful (A)

Calm (ES)

Relaxed (ES)

Sluggish (F)

Figure 1 shows a PDA screenshot of mood items as seen by the participants.

**Figure 1.**


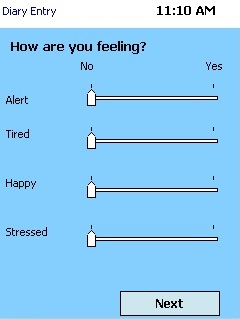


**Work Stress determinants PDA items**

All items refer to last 10 minutes. Analogue items were scored from 0-100. Binary item radio buttons were labelled ‘Yes’ and ‘No’. JCQ = items adapted from Job Content Questionnaire; ERI = items adapted from Effort-Reward Imbalance Questionnaire.

**Analogue scales (summary measures of Demand, Control, Effort, Reward):**

“Think about mental and physical activity in the past 10 minutes …”

• Work has been demanding

• I have put in a lot of effort

• Had control over work

• Work has been rewarding

Figure 2 shows a PDA screenshot of analogue work stress determinants scales, as seen by the participants.

**Figure 2.**


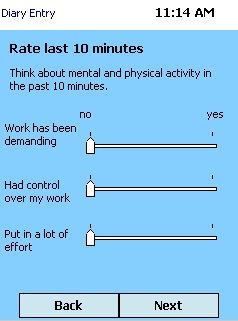


**Binary items:**

*Demand*

“In the past 10 minutes work has required that I…”

• Work Hard (JCQ)

• Work Fast (JCQ)

• Do too much (JCQ)

• Were you interrupted during your main activity in the last 10 mins?

• Enough resources available?

• Enough time available?

*Effort*

“In the past 10 minutes I have…”

• Been under constant pressure (ERI)

• Had a lot of responsibility (ERI)

• Been under a lot of physical demand (ERI)

• Were you interrupted during your main activity in the last 10 mins?

*Control*

“In the last 10 minutes work has…”

- Required a high level of skill (JCQ)
- Allowed me to make the main decisions about what I did (JCQ)
- Allowed me a lot of say in what I did (JCQ)

*Reward*

“In the last 10 minutes my work has been …”

• Appreciated

• Valued

• Respected (ERI)

*Additional items*

“In the last 10 minutes …”

- I would have liked more control of my work
- Work has been challenging

Figure 3 shows a PDA screenshot of binary work stress determinants scales, as seen by the participants.

**Figure 3.**


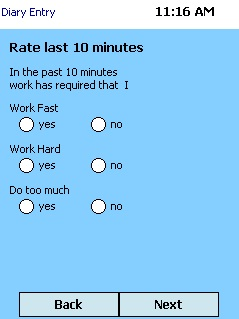


**Nursing task PDA items**

Items were taken from the Work Observation Method By Activity Timing (Westbrook & Ampt, 2009).

Main activity was identified using a mutually exclusive list of work tasks:

- Direct care
- Indirect care
- Medication
- Documentation
- Professional Communication
- Ward related
- In transit
- Supervision
- Social/Break
- Other

Figure 4 shows a PDA screenshot of nursing task items as seen by the participants. There are also items assessing “Who else was involved” and any “Tools/Equipment” used which do not relate to the issues examined in this paper.

**Figure 4.**


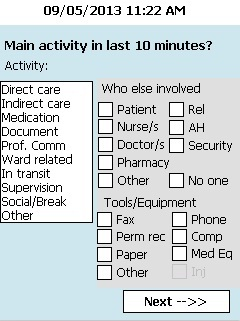

Supplement: Supplementary file 1 — Supplementary Material on PDA items to accompany: “Stressors, appraisal of stressors, experienced stress and cardiac response: a real-time, real-life investigation of work stress in nurses.” (DOCX 211 kb) [file 12160_2015_9746_MOESM1_ESM.docx]
