# Supplementary material for: Stressors, Appraisal of Stressors, Experienced Stress and Cardiac Response: A Real-Time, Real-Life Investigation of Work Stress in Nurses
Source: Ann Behav Med. 2015 Nov 25;50:187–97. doi: 10.1007/s12160-015-9746-8 (PMC4823345; doi:10.1007/s12160-015-9746-8)
Supplement: Supplementary file 2 — Supplementary material – WOMBAT training manual to accompany: “Stressors, appraisal of stressors, experienced stress and cardiac response: a real-time, real-life investigation of work stress in nurses.” (DOC 6.02 mb) [file 12160_2015_9746_MOESM2_ESM.doc]

Appendix 2. Supplementary material – WOMBAT training manual to accompany:

“Stressors, appraisal of stressors, experienced stress and cardiac response: a real-time, real-life investigation of work stress in nurses.”

Training Booklet

**How to describe your main nursing activity on our hand held computers**

When taking part in the study you will be asked to indicate your main nursing activity approximately every 90 minutes on a small handheld computer.

The computer programme we use splits nursing tasks into 10 categories of activity. This booklet has been designed to provide you with training so you are aware of these categories as they may be slightly different to the ones you use in a normal work setting. **Please follow the instructions to fill out this booklet and return it in the addressed envelope along with your consent form and questionnaires.**

*For any questions about this booklet contact Cheryl Bell, email:* [*cheryl.bell@abdn.ac.uk*](mailto:cheryl.bell@abdn.ac.uk)*, tel: 01224438098*

Below is a screenshot from the handheld device with all 10 nursing task categories on the left hand side (figure 1). Attached to the device there will be a ‘glossary’ giving a short definition of each category (figure 2).

Please take a moment to familiarise yourself with the categories and their brief definitions.

Figure 1. Task categories Figure 2. Glossary of brief definitions


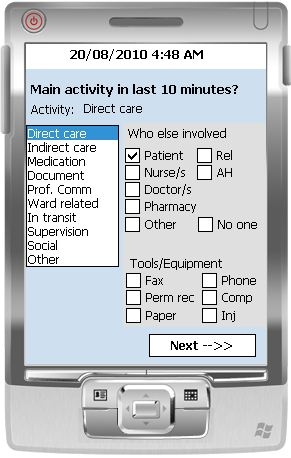


- **Direct Care**: activity directly related to patient care
- **Indirect Care**: activity indirectly related to patient care
- **Medication**: activity that relates to medication for a particular patient
- **Document**: recording of patient information on paper or computer
- **Prof Comm**: work-related discussion with other staff
- **Ward related**: activity which relates to the running of the ward in general that is not related to direct or indirect individual patient care
- **In transit**: work related movement between patients and between tasks
- **Supervision**: active supervision or teaching of another staff member or student
- **Social**: social or personal activity or discussion
- **Other**: tasks not covered above

We will now provide you with more detailed definitions of each category. **In each example, we ask you to draw a line to match each definition to the most appropriate photograph.**

**Direct care:** activity directly related to patient care.

**Includes**: nursing procedures (e.g. catheterisation), bathing, bed making, transporting patient, communicating with patient

**Excludes**: medication, documenting, planning care and communicating with staff

**Medication:** activity that relates to medication for a particular patient.

**Includes**: order, prep, clarify, check, administer, chart, discuss and review

**Please draw a line to match each definition to the most appropriate photograph.**


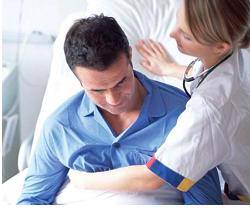

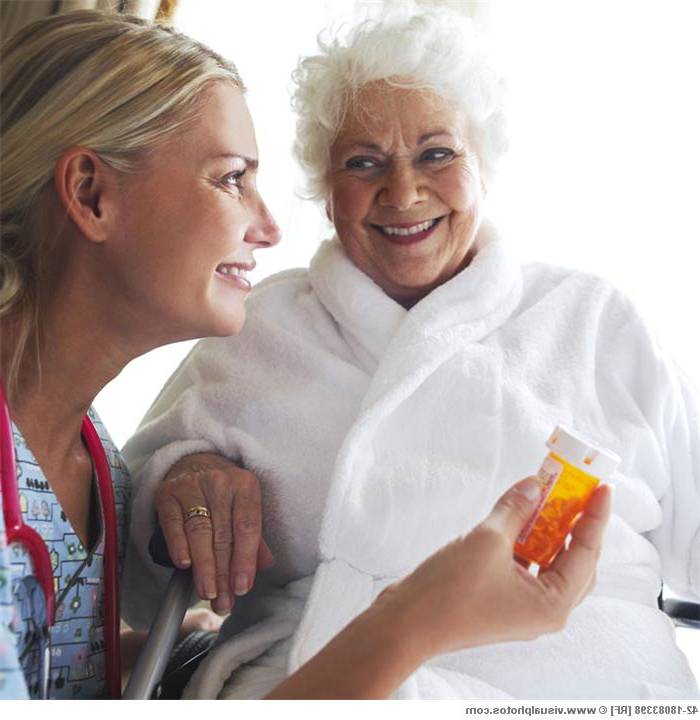


**Indirect care:** activity indirectly related to patient care.

**Includes**: reviewing documents, planning care, ordering tests/diet, retrieving info, checking results, washing hands, gathering/returning equipment, cleaning up

**Excludes**: medication, documenting patient notes, communicating with staff, patient or relatives

**Document:** recording of patient information on paper or computer

**Includes**: documentation on patient care plans

**Excludes**: medication chart documentation

**Draw a line to match each definition to the most appropriate photograph.**


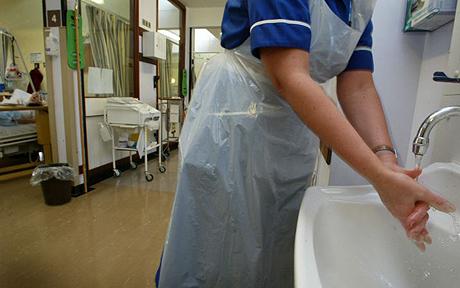

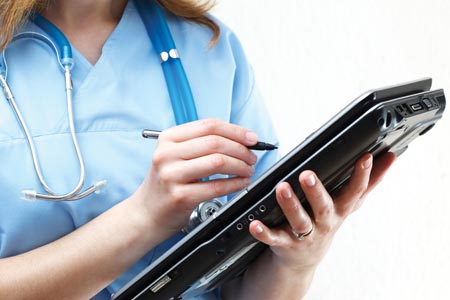


**Professional Communication:** any work related discussion with another staff member

**Includes**: handover, requesting medical or nursing review, planning care staff member

**Excludes**: medication related discussion, communication with patient or relative

**Ward related:** activity which relates to the running of the ward that is not related to direct or indirect patient care

**Includes**: bed allocation, rosters, coordinating staff, meetings, DD checking, ward/stock orders

**Excludes**: handover

**Draw a line to match each definition to the most appropriate photograph.**

**
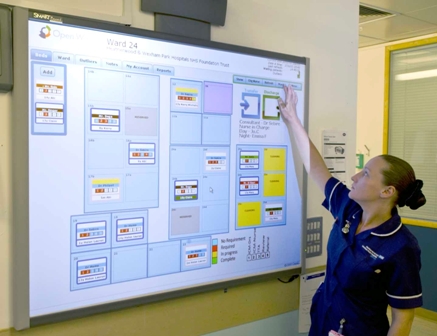

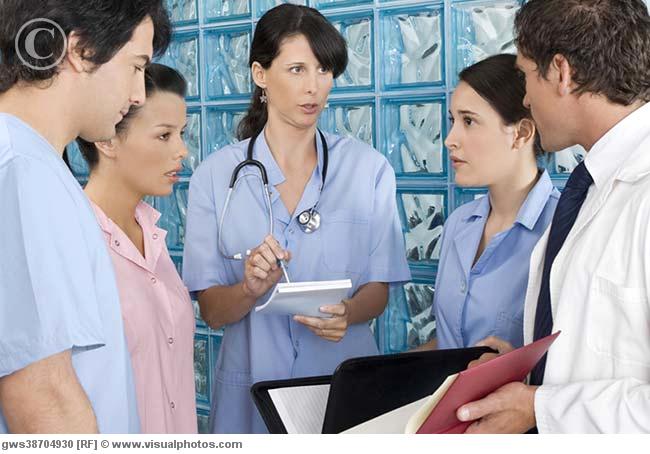
**

**Document:** recording of patient information on paper or computer

**Includes**: documentation on patient care plans

**Excludes**: medication chart documentation

**Indirect care:** activity indirectly related to patient care.

**Includes**: reviewing documents, planning care, ordering tests/diet, retrieving info, checking results, washing hands, gathering/returning equipment, cleaning up

**Excludes**: medication, documenting patient notes, communicating with staff, patient or relatives

**Medication:** activity that relates to medication for a particular patient.

**Includes**: order, prep, clarify, check, administer, chart, discuss and review

**Draw a line to match each definition to the most appropriate photograph.**


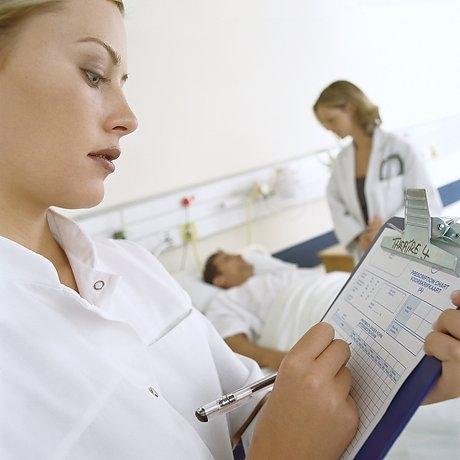

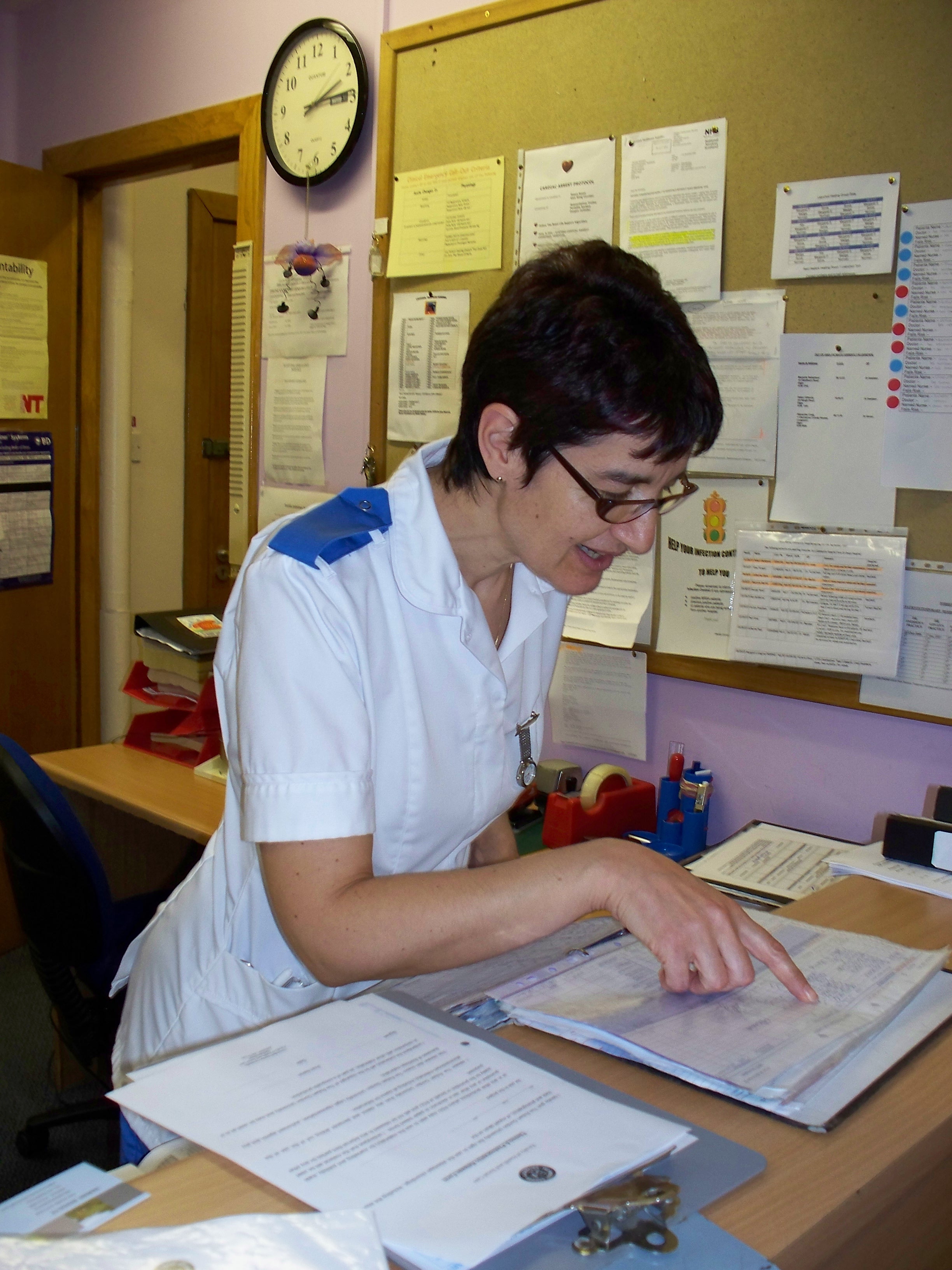


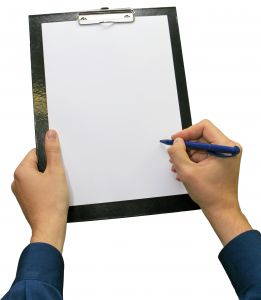


**Please write a letter next to each definition to match it to the most appropriate photo.**

**Professional Communication**

**Ward related**

**Document**

**Indirect Care**

**Medication**

**Direct Care**

**
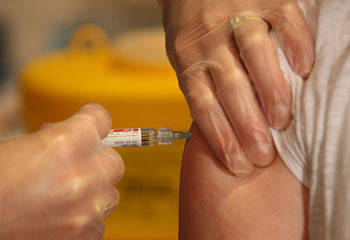
**

**
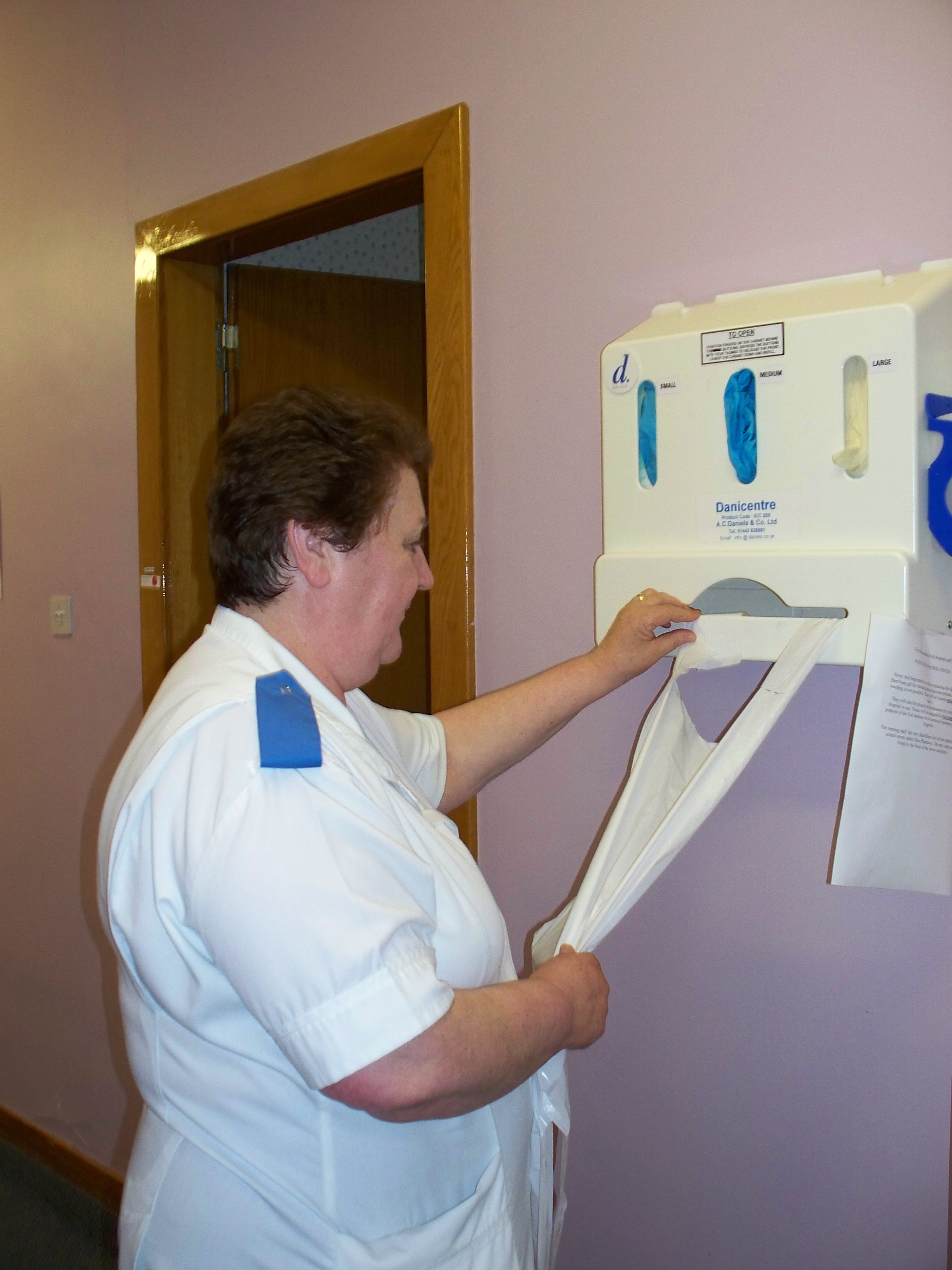

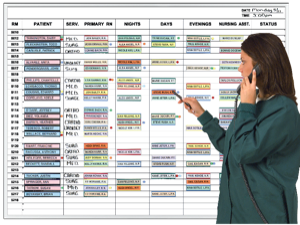

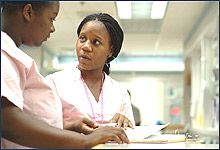

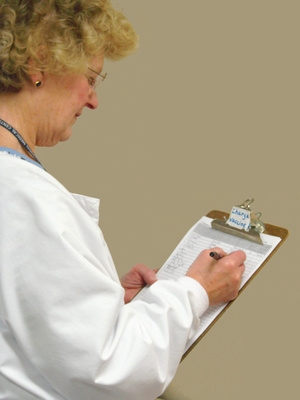

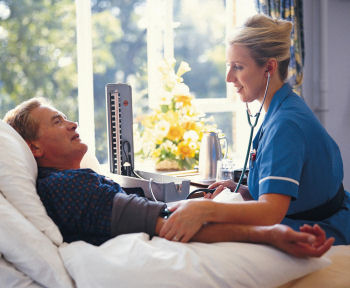
**

A

B

C

E

F

D

[Feedback for page 7 – A: prof comm., B: direct care, C: medication, D: document, E: ward related, F: indirect care]

**In transit:** work related movement between patients and between tasks

**Includes**: movement when a nurse exits a patient room

**Excludes**: movement between patients in a shared room, movement within a single room

**Supervision:** active supervision or teaching of staff or students

**Social:** any social or personal activity or discussion.

**Includes**: personal phone calls, tea, personal breaks

**Draw a line to match each definition to the most appropriate photograph.**

**
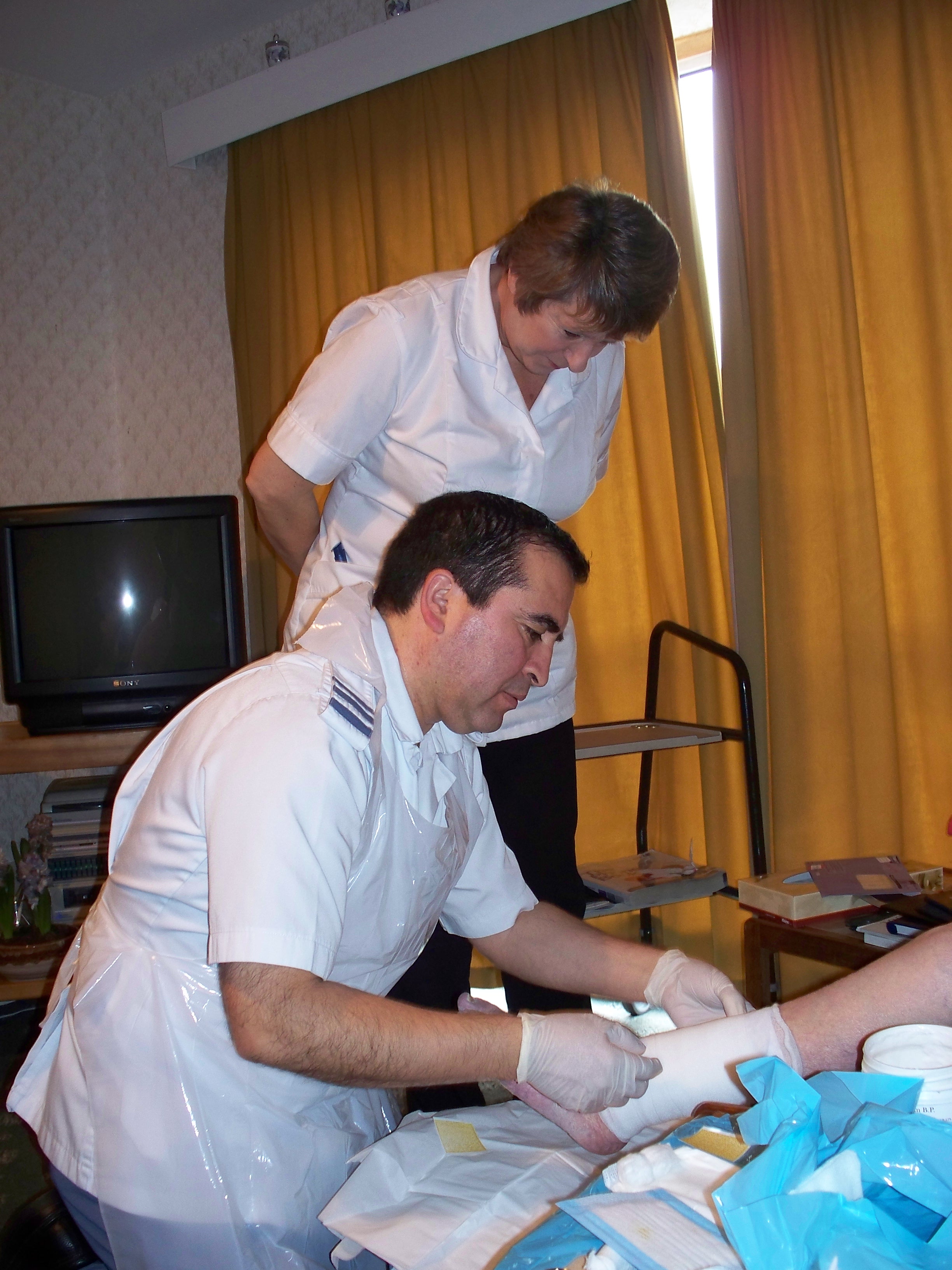
**


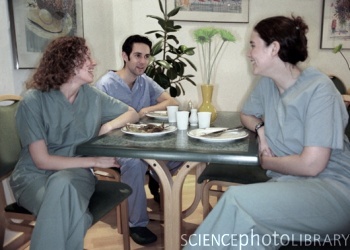

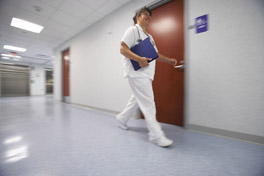


**Write a letter next to each definition to match it to the most appropriate photo**

**Professional Communication**

**Document**

**In Transit**

**Supervision**

**Social**


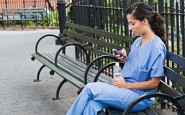


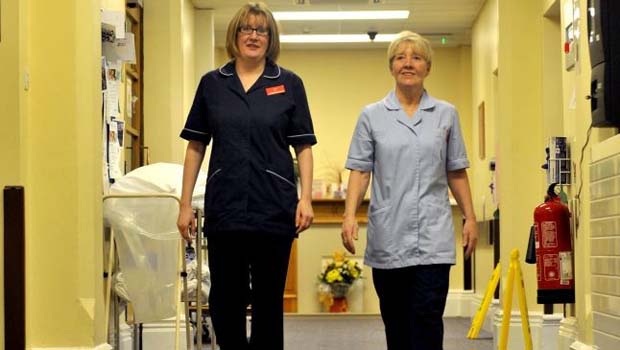


D

C


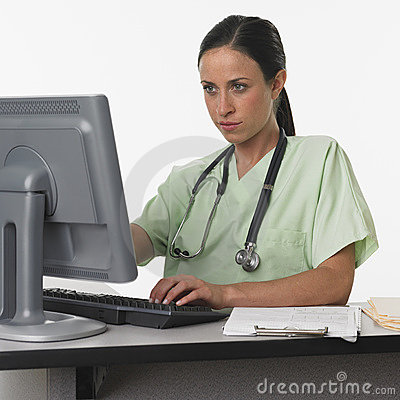

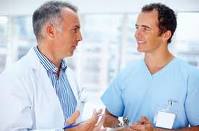

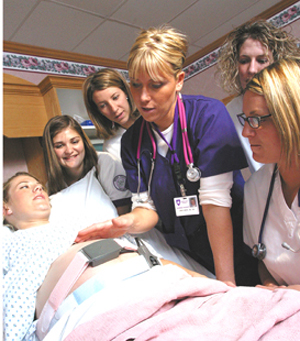


A

E

B

**Write a letter next to each definition to match it to the most appropriate photo**

**Ward related**

**Indirect Care**

**Medication**

**Direct Care**


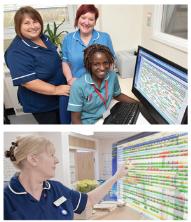


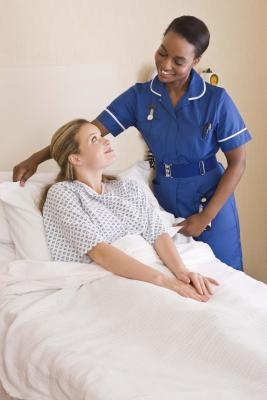


G


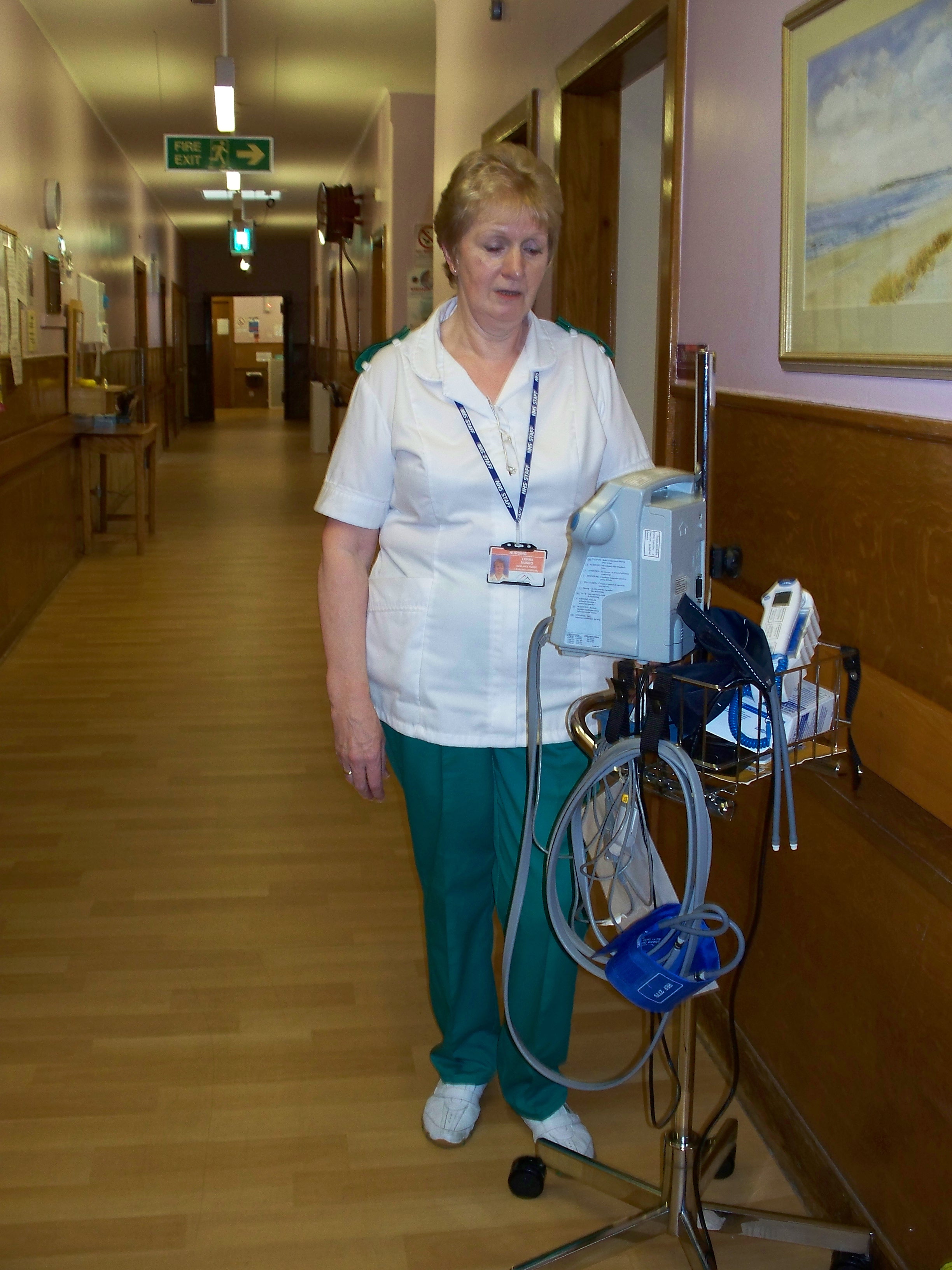


F

I


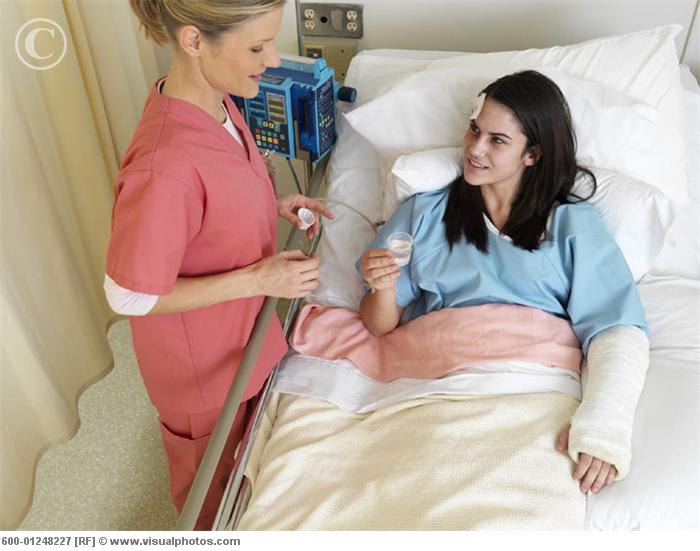


H

**Please return this booklet in the addressed envelope provided.**

Feedback for pages 9 and 10 will be given by the Research Assistant on the day of your participation.

Thank you for your cooperation.
