# Supplementary material for: Stressors, Appraisal of Stressors, Experienced Stress and Cardiac Response: A Real-Time, Real-Life Investigation of Work Stress in Nurses
Source: Ann Behav Med. 2015 Nov 25;50:187–97. doi: 10.1007/s12160-015-9746-8 (PMC4823345; doi:10.1007/s12160-015-9746-8)
Supplement: Supplementary file 3 — Supplementary Material on effects of work tasks to accompany: “Stressors, appraisal of stressors, experienced stress and cardiac response: a real-time, real-life investigation of work stress in nurses.” Complete versions of Tables 3 and Table 6 in the paper showing the estimated beta weights and SE for the work tasks. In all cases direct patient care was used as the reference category (DOCX 17.8 kb) [file 12160_2015_9746_MOESM3_ESM.docx]

Appendix 3. Supplementary Material on effects of work tasks to accompany:

“Stressors, appraisal of stressors, experienced stress and cardiac response: a real-time, real-life investigation of work stress in nurses.”

Complete versions of Tables 3 and Table 6 in the paper showing the estimated beta weights and SE for the work tasks. In all cases direct patient care was used as the reference category.

**Table 1 (expanded version of Table 3): Demand Control and Effort Reward Imbalance Model** for **physiological measures:** results showing estimated beta weights (standard error) for fixed effects and variances for random effects with and without allowing for work tasks

|  | **Demand Control model** | | | **Effort Reward Imbalance Model** | | |
| --- | --- | --- | --- | --- | --- | --- |
|  |  | **Heart Rate** | **Heart rate** *allowing for work tasks* |  | **Heart Rate** | **Heart rate** *allowing for work tasks* |
| **Fixed effects** | |  |  |  |  |  |
|  | Intercept | 83.86 (1.19) | 84.26 (1.21) |  | 83.80 (1.16) | 84.71 (1.18) |
|  | Shift (1 or 2) | -0.10 (0.59) | -0.17 (0.58) |  | -0.16 (0.61) | -0.23 (0.59) |
|  | Time into shift (hours) | -0.31 (0.05 | -0.27 (0.05) |  | -0.30 (0.05) | -0.26 (0.05) |
|  | Linear effect over period before diary entry. | -0.46 (0.07) | -0.46 (0.07) |  | -0.46 (0.07) | -0.46 (0.07) |
|  | Quadratic effect over period before diary entry | -0.03 (0.01) | -0.03 (0.01) |  | -0.03 (0.01) | -0.03 (0.01) |
|  | Accelerometer measure of activity | 1.69 (0.02) | 1.69 (0.02) |  | 1.69 (0.020 | 1.69 (0.02) |
|  | Indirect care |  | -0.60 (0.59) |  |  | -0.68 (0.60) |
|  | Medication |  | -1.65 (0.58) |  |  | -1.60 (0.58) |
|  | Professional Communication |  | -1.48 (0.70) |  |  | -1.69 (0.71) |
|  | Documentation |  | -4.09 (0.64) |  |  | -4.43 (0.64) |
|  | In transit |  | 5.63 (2.46) |  |  | 5.80 (2.47) |
|  | Social/break |  | -0.66 (0.68) |  |  | -1.17 (0.72) |
|  | Other |  | -2.01 (1.93) |  |  | -2.47 (1.95) |
|  | Supervision |  | -5.00 (3.28) |  |  | -4.93 (3.29) |
|  | Ward related |  | -1.27 (0.77) |  |  | -1.60 (0.78) |
| **Between persons** | **Demand** | 2.86* (1.11) | 2.88* (1.11) | **Effort** | 3.25* 1.40) | 3.24* (1.40) |
|  | **Control** | 2.32 (2.11) | 2.29 (2.11) | **Reward** | -0.09 (1.24) | -0.15 (1.24) |
|  | **Demand x Control** | 0.08 (2.15) | 0.14 (2.15) | **Effort x Reward** | 0.54 (1.50) | 0.55 (1.50) |
| **Within persons** over time | **Demand** | 0.84* (0.11) | 0.70* (0.13) | **Effort** | 0.86* 0.14) | 0.68* (0.15) |
|  | **Control** | 0.34 (0.23) | 0.35 (0.22) | **Reward** | 0.0 (0.17) | -0.07 (0.17) |
|  | **Demand x Control** | 1.01 (0.13) | 0.06 (0.13) | **Effort x Reward** | 0.20 (0.12) | 0.19(0.12) |
| **Random effects** | |  |  |  |  |  |
|  | Person | 104.76 (16.07) | 105.9 (16.21) |  | 106.29 (16.34) | 107.5 (16.41) |
|  | Shift | 11.04 (2.40) | 10.48 (2.29) |  | 11.68 (2.50) | 10.80 (2.36) |
|  | Time into shift | 31.00 (1.57) | 29.50 (1.52) |  | 31.35 (1.58) | 29.71 (1.53) |
|  | Period before diary entry | 30.97 (0.50) | 30.98 (0.50) |  | 30.96 (0.50) | 30.97 (0.50) |
|  | Alpha (autocorrelation) | 13.34 (0.44) | 13.35 (0.44) |  | 13.32 (0.44) | 13.34 (0.44) |

Alpha is a time series parameter: the covariance between two observations t time units apart is alpha×1/t

**Table 2 (expanded version of DC section of Table 6): Demand-Control Model** for **experienced stress, affect and fatigue:** results showing estimated beta weights (standard error) for fixed effects and variances for random effects allowing for work tasks.

|  |  | **Experienced Stress** | **Affect** | **Fatigue** |
| --- | --- | --- | --- | --- |
| **Fixed effects** | |  |  |  |
|  | Intercept | 28.00 (1.47) | 81.98 (1.44) | 19.38 (1.76) |
|  | Shift (1 or 2) | -1.06 (0.97) | -2.27 (1.09) | 0.05 (1.18) |
|  | Time into shift (hours) | 0.31 (0.12) | -0.34 (0.09) | 2.04 (0.13) |
|  | Indirect care | 0.27 (1.18) | -1.04 (0.83) | 1.77 (1.04) |
|  | Medication | -4.10 (1.14) | 1.61 (0.83) | 0.09 (0.99) |
|  | Professional communication | 1.92 (1.40) | -1.86 (0.99) | 3.62 (1.24) |
|  | Documentation | -2.15 (1.26) | -0.54 (0.88) | 2.19 (1.10) |
|  | In transit | 17.85 (4.89) | -3.27 (3.43) | -4.05 (4.28) |
|  | Social/break | -1.14 (1.33) | 0.17 (0.93) | 1.51 (1.15) |
|  | Other | -0.60 (3.80) | -3.31 (2.66) | 6.29 (3.32) |
|  | Supervision | -10.75 (6.57) | 3.91 (4.62) | 1.82 (5.82) |
|  | Ward related | -0.41 (1.53) | -1.64 (1.08) | 0.69 (1.36) |
| **Between persons** | **Demand** | 5.21* (1.16) | -0.49 (1.21) | 0.06 (1.46) |
|  | **Control** | -6.93* (2.20) | 6.41* (2.29) | -6.08* (2.76) |
|  | **Demand x Control** | 4.13(2.29) | -3.29 (2.39) | 3.38 (2.87) |
| **Within persons** over time | **Demand** | 2.75* (0.25) | -1.19* (0.18) | -0.50 (0.23) |
|  | **Control** | -2.03* (0.45) | 1.82* (0.32) | -1.08* (0.40) |
|  | **Demand x Control** | -0.45 (0.25) | 0.43 (0.18) | 0.02 (0.22) |
| **Random effects** | |  |  |  |
|  | Person | 99.29 (17.62) | 103.83 (19.36) | 158.16 (27.38) |
|  | Shift | 2.20 (7.79) | 28.64 (8.60) | 0 (0) |
|  | Time into shift | 176.44 (8.94) | 96.57(5.08) | 191.45 (9.21) |
|  | Alpha (autocorrelation) | 40.39 (8.55) | 33.64 (4.67) | 93.45 (7.88) |

Alpha is a time series parameter: the covariance between two observations t time units apart is alpha×1/t

**Table 3 (expanded version of ERI section of table 6) : Effort Reward Imbalance Model** for **experienced stress, affect and fatigue:**  results showing estimated beta weights (standard error) for fixed effects and variances for random effects allowing for work tasks.

|  |  | **Experienced Stress** | **Affect** | **Fatigue** |
| --- | --- | --- | --- | --- |
| **Fixed effects** | |  |  |  |
|  | Intercept | 28.05 (1.48) | 81.81 (1.26) | 19.34 (1.51) |
|  | Shift (1 or 2) | -1.22 (0.96) | -2.26 (1.05) | 0.12 (1.17) |
|  | Time into shift (hours) | 0.32 (0.12) | -0.34 (0.09) | 2.09 (0.13) |
|  | Indirect care | -0.42 (1.20) | 0.01 (0.83) | 1.0 (1.05) |
|  | Medication | -3.61 (1.15) | 1.42 (0.80) | 0.27 (0.99) |
|  | Professional communication | 1.27 (1.41) | -0.88 (0.99) | 2.83 (1.24) |
|  | Documentation | -4.04 (1.28) | 1.01 (0.89) | 0.97 (1.11) |
|  | In transit | 18.22 (4.94) | -2.98 (3.42) | -4.29 (4.28) |
|  | Social/break | 1.43 (1.43) | 1.59 (0.99) | -0.46 (1.23) |
|  | Other | 1.41 (3.87) | -1.4 (2.68) | 4.34 (3.35) |
|  | Supervision | -10.86 (6.63) | 4.42 (4.60) | 2.03 (5.81) |
|  | Ward related | -1.89 (1.55) | 0.15 (1.09) | -0.57 (1.37) |
| **Between persons** | **Effort** | 6.09* (1.52) | -1.10 (1.36) | -0.55 (1.58) |
|  | **Reward** | -5.13* (1.33) | 6.86* (1.19) | -8.56* (1.39) |
|  | **Effort x Reward** | 1.14 (1.61) | -2.07 (1.44) | 3.06 (1.68) |
| **Within persons**over time | **Effort** | 3.20* (0.31) | -1.07* (0.22) | -0.20 (0.28) |
|  | **Reward** | -1.98* (0.35) | 2.17* (0.25) | -1.47* (0.31) |
|  | **Effort x Reward** | -0.59* (0.24) | 0.81* (0.17) | -0.03 (0.21) |
| **Random effects** | |  |  |  |
|  | Person | 111.04 (19.01) | 80.31 (15.67) | 111.85 (20.69) |
|  | Shift | 0 (0) | 23.64 (7.90) | 0 (0) |
|  | Time into shift | 180.82 (7.86) | 96.32 (5.07) | 187.97 (9.02) |
|  | Alpha (autocorrelation) | 42.76 (7.05) | 33.80 (4.65) | 90.05 (7.74) |

Alpha is a time series parameter: the covariance between two observations t time units apart is alpha×1/t
